# Supplementary material for: Are carbon emissions trading and green financial instruments synergistic? -Comprehensive quantitative research based on content analysis
Source: PLoS One. 2024 Mar 7;19(3):e0298601. doi: 10.1371/journal.pone.0298601 (PMC10919601; doi:10.1371/journal.pone.0298601)
Supplement: S1 Data — (ZIP) [file pone.0298601.s001.zip › Data/地级市Z_F工作报告.zip!/║┌┴·╜¡/║┌║╙╩╨/└┤╫╘╣1⁄2╓┌║┼í╛┬φ┐╦╩2╛▌═°í┐_2018.docx]

各位代表：

现在，我代表市人民政府，向大会作工作报告，请予审议，并请列席会议的同志提出宝贵意见。

一、2017年工作回顾

2017年是全党上下、全国人民喜迎党的十九大胜利召开的政治之年，是落实习近平总书记重要讲话精神、推动黑河新一轮振兴发展的定向布局之年，也是在省委省政府和市委坚强领导下、新一届政府的开局奋进之年。面对新市情、新机遇、新挑战，我们紧紧围绕省市党代会部署，坚持稳增长、促改革、调结构、惠民生、防风险，努力破解制约发展的主要矛盾和关键问题，较好地完成了市六届人大一次会议确定的主要任务目标。预计地区生产总值增长6.5%，固定资产投资增长5%，社会消费品零售总额增长10%，公共财政收入增长8.3%，城镇、农村常住居民人均可支配收入均增长8%，万元地区生产总值综合能耗下降3%。

(一)做好“三篇大文章”，深化供给侧结构性改革，发展的内生动力显著增强。

坚持一产抓融合，发挥高纬寒地生态优势，促进传统农业向优质绿色的现代农业转变，农业增加值增长6.3%。以科技为引领，引进中国工程院两位院士领衔的专家团队，分别建立我国最北端大豆和汉麻院士工作站，提升优势农产品的核心竞争力。大力发展特色种植，高蛋白有机大豆、无腥味大豆、专用玉米、鲜食玉米、功能水稻等种植面积增长23.6%，亩效益高于普通作物20%以上。加快现代畜牧业发展，与中国农业大学、省农科院畜牧研究所合作组建黑河马繁育、安格斯肉牛养殖专家工作站，孙吴丁子山养殖基地成为国家级畜禽养殖标准化示范场。加快发展林产经济，小浆果、北药面积达到27万亩，食用菌达到6000万袋，林业总产值增长15%。拓宽农产品销售渠道，在北京、上海、无锡开设绿色食品专营店，农产品网上销售额达到1.5亿元，嫩江被农业部确定为“保险+期货/期权”大豆价格保险全国唯一试点县。

坚持二产抓提升，培育壮大特色优势产业，规模以上工业增加值增长7%。加快产业项目建设，全市重点产业项目开复工61项、完成投资67亿元，其中省重点开复工22项、完成投资28亿元，OLED有机发光材料等一批发展前景广阔的新项目开工建设。对接一产上项目，加快推进60万吨水稻加工、大豆分离蛋白、桦树茸加工、中药饮片、温氏生猪养殖一体化等项目，向“粮头食尾”“农头工尾”集中发力，延伸农林和畜牧产业链。围绕矿业上项目，新探明的35吨金、32万吨铜、2.2亿吨煤经济价值达千亿元，东安岩金矿、争光岩金矿、北大荒矿泉水开发等推进多年的重点项目建成投产，翠宏山铁多金属矿、富宏煤矿等项目加快推进，矿业增加值占规模以上工业增加值的“半壁江山”。盘活存量上项目，利用多年未投产的合盛多晶硅项目开发高纯荧光粉产品，投资多年未生产的龙江化工新上10万吨生石灰项目投入运营。扩大增量上项目，支持现有企业做大做强，组建市国投集团，为保税物流中心(B型)等项目提供1.7亿元融资支持；广泛招商引资，与月星集团、雏菊机构、中合置业等大企业深度对接，与珠海建立全面对口合作机制，成功举办大黑河岛国际经贸洽谈会，全市落实招商项目179项，到位资金128亿元，增长6%。

坚持三产抓拓展，大力发展新经济、新业态、新模式，服务业增加值增长6.8%。开发全域旅游，推进五大连池火山博物馆、北安宝地温泉酒店等16项重点旅游项目，推出黑龙江游船和五大连池风景区低空飞行、水秀表演等一批新产品，打造“黑河·我的极致远征”自驾游品牌，与春秋旅游集团共同设立华东地区首家黑河旅游体验店入驻上海，大沾河风景名胜区晋升国家级，使我市国家级风景名胜区数量占全省一半。加大营销推介力度，组织中俄全域旅游推介会等各类旅游产品推介19次，黑河的知名度和影响力全面提升，先后荣获“2017最美中国榜国际知名旅游度假城市”“最具投资价值全域旅游目的地奖”和“全域旅游创建典范城市”称号。全年接待旅游者962万人次，旅游收入82亿元，分别增长18%和20%。推动旅游、康养深度融合，接待国内外旅居老人3万余人，其中俄罗斯老人6000余人。试车经济持续升温，一汽大众集团象山试验场建成投入使用，中国汽车工业协会同意授予我市中国寒区试验场基地称号，宝马、奔驰、奥迪等汽车名企汇集黑河，促进了试车经济与旅游产业、现代服务业融合发展。加快发展电子商务，全市实现网络交易额30.2亿元，自建电商平台达到13家，丰泰公司总代理的俄罗斯紫皮糖“双十一”成交150万单，俄罗斯与黑河特色商品体验馆落地珠海，合作区跨境电商产业园被省商务厅评为省级电子商务示范基地。

(二)主动融入“一带一路”开放布局，积极参与“中蒙俄经济走廊”建设，跨境合作平台的地位更加凸显。

政策争取实现重大突破。在黑河设立重点开发开放试验区被纳入国家守边固边指导意见，省政府出台了21条支持政策。跨境经济合作区得到国家商务部大力支持，列入中俄总理会晤议题并与俄方就跨境合作达成一致。边境旅游试验区实施方案已报国务院待批，正与阿州共同推进跨境旅游合作区。保税物流中心(B型)通过国家部委联合审批，完成7个仓库钢结构工程和区内道路建设。积极对接国家兴边富民政策，梳理出12个板块25项重点项目，争取保障性安居工程、重大水利工程等专项资金10.4亿元。在国家和省大力支持下，俄电专属供电区独立定价机制取得重大突破，将促进俄电产业走出困境、转型升级。

跨境基础设施通道建设进展顺利。黑龙江大桥双方同步加快建设，中方超额完成年度计划；启动桥头区建设规划和产业项目招商工作，与月星集团、海航集团、中北科技等多家企业达成合作意向。跨江空中索道前期工作完成。中俄东线天然气管道首条穿江隧道贯通，在黑河设立天然气贸易公司得到中石油大力支持，将进一步助力黑河发展。五大连池德都机场正式通航，创造东北地区首家当年开工、当年竣工的纪录。积极申请开放黑河国际空港并与俄伊尔航空公司签订了旅游包机协议。争取上级支持，五大连池至伊春汤旺河旅游公路已纳入省推进项目。

贸易投资合作积极向好。主动应变，连续4年外贸大幅下滑的趋势得到扭转，边境小额贸易、外贸进口企稳回升，分别增长24%和15.6%。进出口货物和进出境人数分别增长19.6%和4.9%，其中进口木材70万立方米，增长超过一倍。在俄农业种植面积超过130万亩、位列全省第二，回运大豆25万吨，占全省俄进口大豆的60%。阿穆尔—黑河油品储运与炼化综合体项目启动与央企合作洽谈，萨哈(雅库特)油田区块勘探开发完成两个井的钻探工作，境外石化建材、农业畜牧和工业园区均列入省重点园区。

深化对俄人文交流。第八届中俄文化大集首次在北京俄驻华大使馆召开新闻发布会，活动领域从文化艺术展示向高端互动交流、文化贸易、文化产业合作拓展，成为中俄国家级大型文化交流活动。成功举办中俄合作高峰论坛、中俄林业生态建设国际学术论坛、食用大豆产业发展高峰论坛、中俄医科大学联盟健康黑河发展论坛等高端学术活动，与中国社科院边疆研究所和国家发改委、商务部研究院开展战略合作，借助国家智库力量为黑河争取跨境合作政策。支持俄阿州布市成功举办首届阿穆尔经济论坛，形成中俄双方共同推介的联动效应。首次在俄布市开展中俄知识产权文化交流活动，连续四次组队参加俄远东地区鄂温克鄂伦春语言奥林匹克竞赛，举办了横渡黑龙江、中俄中小学校园雪地足球赛、界江国际冰球友谊赛等精品赛事，组建中俄综合国际艺术团联合巡演，开创了“一带一路”沿线国家合作演出新模式。

(三)坚持以人民为中心的发展思想，在增进民生福祉上持续发力，让群众得到更多看得见摸得着的实惠

首次以《民生工作报告》把民生工作摆上突出位置，全市投入财政资金147亿元、撬动社会资本187亿元投向民生领域，116项民生项目建成使用，让更多群众分享发展成果。

坚决打赢精准脱贫攻坚战。市县两级全面落实责任，将工作力量向基层一线倾斜，全市抽调381名干部组成127个驻村工作队开展一对一帮扶。实施汉麻、畜禽养殖、光伏电站等产业扶贫项目237项，引导贫困户发展致富产业。改善生产生活条件，新建维修了131个贫困村的道路、路灯、公厕等基础设施，解决了42个贫困村饮水不安全问题，改造贫困户危房1721户，为贫困人口全部办理了新农合、大病医疗和人身意外伤害保险。省级贫困县孙吴和全市57个贫困村、6559名贫困人口达到摘帽出列标准。

抓住国家三年政策支持的最后一年窗口期，全面实施了黑河城建史上投资最大、受益群众最多的市区棚户区改造工程。年内启动征收4608户，征收面积23.04万平方米，征收完成率94.16%，超额完成省政府下达的改造任务，完成计划118.52%，开工率124.79%，均居全省之首。回迁安置工作同步展开，年内货币化安置1676户，3.1亿元房屋补偿款全部发放到位；采购房源回迁安置324户，实现了当年征收、当年入住；实行产权调换安置847户，投资6100万元开工建设3.4万平方米两个回迁区。通过棚改工作，解决了多年想解决而没有解决的难题，盘活了长期闲置的楼房3.3万平方米，最长达10年之久的668户未回迁居民得到安置，解决了历史遗留的826户回迁居民的不动产权证办理难题，并以此为契机，彻底解决了29个项目、54.4万平方米、4500余户房屋产权办证难题。争取专项资金1.3亿元，用于回迁小区配套设施建设，将极大改善群众的居住条件和生活环境。各县(市)也同步推进棚改，启动征收2137户，征收面积15.43万平方米。

提升城乡居民生活品质。新建北安祥和广场、瑷珲乡野公园等休闲场所12个，增加活动面积33.2万平方米，新增城市绿地94.8万平方米。高水准设计建设黑龙江沿江公园改造工程，克服水利规划调整难、有效工期不足3个月、汛期施工复杂等困难，完成堤顶和广场改造升级8.3万平方米，增加群众休闲健身面积4.6万平方米，马占山公馆、黑河商会两处复古建筑主体完工，修建大黑河岛环岛路5公里、景观路2360米、绿化1.6万平方米，中心广场竣工，体育公园投入使用。统筹堤防建设与市政建设，四岛防护工程全面完工。“三供两治”项目扎实推进，全市新建和改造供水管网17.2公里，市净水厂完成工艺改造；改扩建污水处理厂2座，污水日处理能力15.7万吨，提前三年达到“十三五”期末国家标准；改造老旧供热管网40.3公里，新增供热面积55.7万平方米。市区修建燃气管道23公里，管道天然气正式通气，受益500余户家庭。大力开展城乡环境综合整治，市区清运垃圾4.7万吨，拆除违建仓库、车库等116个，黑河通过国家卫生城复检，五大连池顺利通过国家卫生城技术评审，嫩江、逊克获得国家卫生县城荣誉称号。

生态文明建设取得新成果。历经15年创建，黑河通过省级生态市考核验收。全市造林绿化9万亩，超额完成省下达任务，坤河湿地公园晋升国家级，嫩江科洛火山景区成功创建省级森林公园，全市省级以上森林公园达到6处，逊克成为我省唯一的全国第三批低碳城市试点。建立四级“河长制”，全省最严格水资源管理制度考核我市名列第一。五大连池风景区良好湖泊保护项目顺利实施，获得国家财政部和环保部专项资金2.25亿元；建设农业“三减”示范区170个216万亩，生态红线划定工作位居全省前列；深入推进秸秆综合利用，逊克经验被推广到全省。市区淘汰燃煤小锅炉34台，全市淘汰黄标车2095台，提前完成省定任务，空气质量持续领跑全省，入选“2017中国好空气城市排行榜”。加快美丽宜居乡村建设，4个村被中央文明办评为第五届全国文明村镇，3个民族村获国家民委少数民族特色村镇命名，五大连池镇、赵光镇入选第二批国家级特色小镇。

推进基本公共服务均等化。优先发展教育事业，全市新建改扩建公办幼儿园6所，学前教育三年毛入园率84.8%，超过省定目标4.8个百分点；义务教育标准化学校达到104所，市直、孙吴、逊克和爱辉区义务教育发展基本均衡高水平通过国家验收；高考成绩实现“六连增”。资助2.6万名贫困学生顺利完成学业。医疗服务能力进一步提升，市第一人民医院改扩建和儿科楼主体工程完工，嫩江妇幼保健计生服务中心综合楼投入使用，全市完成4家县级医疗机构和26家贫困村卫生室标准化建设任务。推进文化惠民工程，中心村文化广场达到215个，举办“全省首届社区文化艺术节暨2017年黑河市民文化节”，创编的鄂伦春民族原生态音乐剧获省第六届少数民族文艺会演最高奖，央视春节品牌节目《东西南北贺新春》首次选设黑河分会场，将集中展现我市民族特色、区位优势和多元文化。《瑷珲镇志》《新生村志》纳入国家名镇名村志文化工程，黑河知青博物馆入列国家方志馆分馆。全市新增体育健身场地158处，市区新建滑冰场、冰球场31处，市全民健身中心体育馆、五大连池文体中心建成，逊克全民健身中心开工，举办全国火山雪地徒步、国际火山马拉松等赛事活动120余项，爱辉区、五大连池风景区荣获“全国群众体育先进单位”称号。

织牢织密社会保障网。率先在省内实施失业保险技能提升补贴，城镇新增就业1.6万人，城镇登记失业率3.54%，低于省定标准近1个百分点。社会保险全民参保登记入库162.9万人，基础数据库覆盖全市范围。完成城乡居民基本医疗保险管理体制整合，信息系统率先在全省实现与国家异地就医即时结算平台对接，异地就医结算2221人，极大方便了群众。加快养老机构建设，新增民办养老机构9家，每千名老人拥有养老机构床位45张，超过全省平均水平13张。加大扶老助残力度，发放高龄津贴、贫困失能老年人护理补贴2027万元，为22566名残疾人发放生活和护理补贴2155万元。优抚对象抚恤补助全部达到国家标准，城乡低保、特困人员供养全部达到省定标准，特困供养人员住院自负费用救助比例100%，医疗救助封顶线达到3万元。累计投入3.8亿元救助资金，解决了11万名困难群众的生活、看病难题。

老区、侨务、妇女儿童、民族宗教、人防、防震减灾、档案等工作也取得新成绩。

(四)坚持底线思维，完善社会治理体系，为边城和谐、边境安宁、边疆稳定作贡献

狠抓安全生产责任落实，构建安全风险管控和隐患排查治理双重预防机制，全市未发生较大以上安全生产事故，有力保障了人民群众生命财产安全。狠抓森林防火工作，提前一个月打响春防“四大战役”，连续23个防期未发生重特大森林火灾，实现了不发生人为火灾的历史性突破。

积极推动社会治理重心下移，创新实施社区、社会组织、社会工作“三社联动”治理模式，加快建设智慧社区。高度重视信访工作，扎实开展矛盾大排查、积案大起底、领导大接访“三大行动”，各级领导干部共接待群众3487人次，解决信访突出问题1486件。

推进平安黑河建设，开展社会治安重点地区、公共安全隐患、矛盾纠纷“三项排查”，市区重点区段部位实现监控全覆盖，白天见警徽、晚上见警灯，治安防控成效显著，群众安全感大幅提升。加强国防后备力量建设，积极支持驻黑军警部队改革，巩固双拥模范城创建成果，退役士兵安置遗留问题全部解决，军民融合和军民共建取得新成效，边境管控体系进一步完善，边境秩序保持和谐稳定。

(五)加强政府自身建设，坚决落实市委各项部署，科学化、规范化、机制化水平全面提升

全力推进重点领域各项改革。深化现代农业综合配套改革，土地确权登记进入颁证阶段，北安被农业部确定为全国农村集体产权制度改革试点。市直属国有林场改革实施方案已上报省政府待批。加快财政体制改革，全面实施财政预算绩效管理，国库集中支付动态监控实现全覆盖。率先在全省完成城市综合执法体制改革，集中行使行政处罚权。深化医药卫生体制改革，市域内公立医院药品加成全部取消，医联体建设覆盖所有乡镇，在全省公立医院综合改革考核评估中排名第一。

创新政府管理体制机制，再造工作流程，在已取消和规范证明事项83项的基础上，又取消市本级行政许可事项19项，国家和省取消下放的事项全部落实。提升市综合服务大厅功能，实现政务服务事项标准化，推行“四零”服务承诺制，提高办事效率。实行“多证合一、一照一码”和“两证整合”，新登记市场主体1.5万户，市场主体总数达到8.3万户。坚决落实减免税政策，为企业减负18.4亿元。设立“市长质量奖”，五大连池风景区荣获首届“省政府质量奖”并获全国提名。加强政府立法工作，市人大颁布了有史以来第一部地方实体法规《黑河市市容和环境卫生管理条例》，使城市管理工作有法可依。

全力打造“五型政府”，增强政府执行力和公信力。坚持科学民主决策，主动接受监督，50件人大代表建议、115件政协委员提案全部高质量办结。组建了政府法律顾问团，加强重大决策合法性审查。率先在全省成立开放发展智库联盟，中外专家学者提出各类有价值建议126条，为政府科学决策提供智力支持。全力支持黑河学院发展，开创市校合作共建新模式，推进文体设施共建共享。柔性引进高层次人才，大力培养本土人才，领导干部联系服务专家，32人获国务院特殊津贴、省级优秀中青年专家荣誉。出台市政府工作规则，完善制度，健全机制，政府工作更加规范有序。积极推进“两学一做”学习教育常态化制度化，牢固树立“四个意识”，坚持“五种思维”，增强“八个本领”。深入开展机关作风整顿活动，争做“三个好把式”，形成“五个好作风”，以作风转变促进发展环境优化。严格履行“一岗双责”，政府系统党风廉政建设进一步加强。

各位代表，即将过去的一年，我们在振兴发展的征程中迈出了坚实步伐。这是省委省政府和市委正确领导的结果，是市人大、市政协和社会各界鼎力支持的结果，是全市人民团结一致、共同奋斗的结果。在此，我代表市人民政府，向辛勤工作在各行各业的全市人民，向各级人大代表、政协委员和各民主党派、工商联、无党派人士、人民团体，向关心支持地方工作的中省直单位和解放军、武警官兵，表示衷心的感谢并致以崇高的敬意！

在总结过去一年工作的同时，我们也清醒认识到黑河发展中所面临的困难和挑战。主要是：基础设施建设滞后、高层次人才偏少等瓶颈问题还没有根本解决，加快发展的任务依然艰巨；主导产业层次不高，立市立县大项目不多，新旧动能转换较慢；“一带一路”支点城市的作用还没有充分发挥，对俄合作转型升级面临较大压力；民生领域还有不少短板，城乡功能配套不完善，公共服务能力不强，与群众对美好生活的新期盼还有差距；部分干部思想观念落后，主动服务意识和攻坚克难能力有待提升，发展环境需要进一步优化，等等。对于这些问题，我们将积极应对，扬长补短，全力加以解决。

二、2018年工作安排

2018年是贯彻党的十九大精神的开局之年，是决胜全面建成小康社会、实施“十三五”规划承上启下的关键一年。国家兴边富民、守边固边一系列重大政策密集出台，黑河承接国家大政策将实现历史性突破，黑龙江大桥、跨江空中索道、中俄东线天然气管道等一批跨境项目将在两年内全部建成，俄罗斯全面加快远东地区开放开发步伐和我省与广东省全面对口合作，这些有利因素都将为我市发展提供更加广阔的空间。黑河作为瑷珲—腾冲中国人口地理分界线的端点，解决发展不平衡不充分问题任务重、责任大，我们一定要提高全局站位，主动担当、积极作为，奋力推进经济社会跨越发展，为全国全省发展大局作贡献！

按照市委部署，2018年政府工作的总体要求是：全面贯彻党的十九大精神，以习近平新时代中国特色社会主义思想为指导，按照中央经济工作会议部署，牢牢把握高质量发展这个根本要求，紧紧围绕省委十二届二次全会和市委六届三次全会目标任务，坚定实施绿色崛起、扩大开放、融合发展、创新驱动、差异化发展“五大战略”，坚决打赢解放思想、产业发展、精准脱贫、保障和改善民生、整顿作风优化发展环境“五大攻坚战”，着力构建“十大新体系”，坚决守住发展、生态、民生、安全“四条底线”，加快全面振兴，实现兴边富民，为决胜全面小康、建成富强民主文明和谐美丽的社会主义现代化新黑河打下坚实基础。

全市经济社会发展的主要预期目标是：地区生产总值增长7—10%，固定资产投资增长7%，社会消费品零售总额增长10%，公共财政收入增长6%，对外贸易进出口总值增长6%，城镇、农村常住居民人均可支配收入均增长8%，万元地区生产总值综合能耗下降2%。

为实现上述目标，我们将着力实施振兴发展“八大工程”，全力抓好事关长远“十件大事”。

(一)实施产业项目突破工程，筑牢实体经济根基。坚决打赢产业发展攻坚战，振兴实体经济，培育黑河特色支柱产业。

推进产业项目建设。围绕做好“三篇大文章”，安排重点产业项目75项，年度投资80亿元，其中省重点16项，年度投资27.4亿元。深度开发“原字号”，推进100万吨非转基因大豆综合加工、汉麻纤维加工、杂粮深加工等项目建设，多宝山铜矿二期、苏打水产品、硅藻页岩深加工等项目开工。培育壮大“新字号”，推进OLED有机发光材料、生物质发电、光伏发电等项目建设。改造升级“老字号”，推进文革山铜矿、永发煤矿、山口湖水电增效扩容改造等项目建设。每个县(市、区)盘活3—5户停产半停产企业，让老企业重新焕发生机。扶持企业发展壮大，规模以上工业增加值增长7%。

全力扩大项目增量。围绕国家产业布局扩增量，抓住国家扩大生物燃料乙醇生产的机遇，加强与国投生物科技等央企合作，全力推进玉米燃料乙醇项目。围绕招商引资扩增量，突出以商招商、定向招商、产业链招商，赴京津冀、长三角、珠三角等重点地区开展推介，争取中铁建集团、东方园林公司招商项目落地，招商到位资金增长6%以上。围绕对口合作扩增量，深化与珠海对口合作，全面落实两地签署的框架协议，建立长期稳固合作机制，推动开通两地航线，加强绿色农产品加工销售、旅游康养、园区共建等领域合作。

提升服务保障水平。强化项目跟踪服务，集中会办解决立项审批、落地开工、投产达效难题。提升国网电力保障能力，加快推进500千伏黑河换流站扩建工程，争取开工建设北安500千伏输变电工程。出台加强金融工作意见，助力实体经济发展。扶持更多优质企业挂牌上市，增加直接融资。梳理园区土地、厂房、设备等存量资产，探索采用“飞地经济”模式共建产业园区。

(二)实施乡村振兴战略工程，加快打造现代农业新体系。推进农业供给侧结构性改革，提升一二三产深度融合水平，加快农业农村现代化步伐。

加快发展特色农业。坚持市场导向与科学轮作相结合，大力发展生态绿色特色种植，建设大豆、小麦、玉米、水稻等八大特色基地，推动农业发展从增产向提质转变。打造绿色种植示范区，优质品种统供率达到50%以上，建设绿色大豆高产高效攻关核心区20万亩，辐射带动100万亩，绿色有机食品认证面积达到350万亩以上。提升农业种植科技化水平，加快省级“互联网+农业”高标准示范基地建设，完善物联网、远程监控等功能。发展“两牛一猪一马”，推进北安温氏生猪、孙吴安格斯肉牛等标准化养殖场建设，打造全国高端畜产品供给基地。扩大冷水鱼、稻蟹综合养殖规模，增加名优新品种，拉动渔业经济增长。

延伸农林产业链。坚持以“粮头食尾”“农头工尾”为抓手，培育大豆、汉麻、玉米、肉类加工等十亿级产业，努力把农产品精深加工打造成重要主导产业。加快农作物下游产品研发，发挥顶级科研团队和院士工作站技术引领作用，推进大健康系列大豆科技成果转化，开发以大豆为原料的保健、时尚、休闲食品。加快孙吴汉麻产业园区建设，发展纺织、制药、装饰材料、保健食品等汉麻下游产品，打造从种植优势到产业优势的全产业链。拉长小浆果和北药加工链，提升林产品附加值。推进现代农业产业园建设，构建北安—五大连池—孙吴—爱辉区、逊克—孙吴—嫩江两条功能食品加工走廊。

强化农产品市场营销。组建市级销售公司，做大做强黑河大豆网、北安通、嫩商网，支持瑷珲e购与中国供销集团战略合作，全市利用电商平台线上农产品销售率达到10%。鼓励村级集体经济组织兴办各类新型农业经营主体，与主销区和企业建立长期稳定合作关系，接入各类电商平台。加大宣传营销力度，聘请顶级团队提高产品包装设计档次，完善孙吴山珍产品交易市场，组织开展农产品进北京、上海、珠海活动，设立绿色农产品旗舰店，做大黑河大豆、十六膳等品牌，降低卖原粮、大吨粮比例，让黑河的优质农产品卖出好价钱。

确保精准脱贫攻坚战取得全胜。全面落实脱贫攻坚责任制，各负其责，合力攻坚，发挥驻村工作队作用，与县、乡、村干部共同制定发展规划，培育“一村一品”专业村和特色种植养殖专业户，因地制宜发展特色致富项目，激发贫困人口内生动力。建立脱贫长效机制，落实好“两不愁三保障”政策，加快老区脱贫步伐，确保贫困群众一个不掉队，全市贫困村、贫困人口全部脱贫摘帽。实施农村人居环境整治三年行动，继续推进农村危房改造，开展“四好农村路”建设。加快培育特色小镇，以各县(市、区)中心村、抵边村为重点，建设美丽宜居乡村，推动城乡协调发展。

(三)实施对俄合作升级工程，打造“一带一路”重要支点。坚定不移实施扩大开放战略，推动中俄区域一体化进程，构建全方位对外开放新格局。

推进跨境基础设施互联互通。加快黑龙江大桥建设，确保跨江空中索道开工。加强与月星集团、海航集团等企业合作，组建桥头区开发建设主体，推进桥头区一期启动区建设，基础设施和口岸联检区开工。扩建黑河瑷珲机场停机坪，争取开通赴俄临时包机，五大连池德都机场力争开通新航线，嫩江墨尔根通用机场实现通航，启动逊克通用机场项目前期。做好中俄东线天然气管道工程保障，黑河输气首站开工。积极引进主体，确定北黑铁路改造升级最佳合作形式，完成嫩江地铁北站建设，做好北五铁路、黑河至乌伊岭、黑河至韩家园子铁路前期工作。争取黑河口岸连通工程列入交通部“十三五”规划，完成开工前各项准备，协调推进我省北部旅游公路前期工作。

深化对俄贸易投资合作。稳定进出口主营骨干品种，扩大风电设备、硅基材料等地方工业品出口，电力、木材等能源资源商品进口，争取获批进口水产品指定口岸资质。支持企业扩大对俄投资，参与俄资源能源开发，争取阿穆尔—黑河边境油品储运与炼化综合体、阿州锑矿开采项目开工。加快合作区汽车零配件产业园建设，建成进口机电设备及车辆国检试验区，继续推进布市利源达专用车制造厂项目，推动形成跨境机电产业链。扩大对俄农林合作规模，引导企业由境外农业种植、森林采伐向农林产品深加工发展，逐步实现生产、加工、贸易一体化。充分发挥保税物流中心(B型)带动作用，打造现代跨境商贸物流集散中心和进出口生产加工基地。加快境外园区建设，支持企业开拓俄罗斯市场。

加强人文领域交流。扩大“一带一路”合作领域，建设“冰上丝绸之路”合作平台。办好第九届中俄文化大集，打造国际性、专业化、开放式的高端平台。提高中俄边境城市展览会层次、规模和水平。深化对俄科技、教育、医疗、体育等领域交流，加强与俄远东城市联盟合作，办好中俄沿边城市联盟首届峰会、青少年国际夏令营、界江国际冰球友谊赛等活动和赛事，深入双方腹地开展文化交流演出和民间手工艺品展览，开展与俄远东地区少数民族文化交流活动，打造中俄人文交流示范区。

(四)实施新兴业态引领工程，激发经济发展新动能。坚定实施创新驱动发展战略，大力发展新经济、新业态、新模式，加速形成新的经济增长点。

培育全域旅游品牌。编制市区5A级景区旅游规划，创建全域旅游示范区，建设旅游强市。鼓励和引导社会资金投入，建立多元化投入机制。推进黑龙江游船、五大连池火山博物馆、瑷珲乡野公园等项目，谋划建设孙吴、逊克自驾车营地，争取开通8座以下小型车辆跨境自驾游。发展智慧旅游，推进五大连池风景区全自动化管理服务，实现全市重点景区免费WiFi全覆盖。加大旅游产品供给，大力发展研学游、乡村游、工业游和定制旅游。组织开展系列营销活动，叫响“黑河？我的极致远征”自驾游和“亲亲的黑河”研学游特色品牌，进一步增强黑河旅游吸引力。

扩大康养产业规模。推动养老、健康、食品药品产业融合发展，提升候鸟老人旅居养老和跨国互动规模。健全完善养老服务新模式，谋划推进中俄康养中心、五大连池风景区人民医院医养合作等项目，吸引更多的民间资本投资健康养老产业。探索社区养老服务信息平台建设，开展居家养老服务，满足老年人多样化需求。强化公办养老机构兜底作用，提高失能养护服务保障能力，完成嫩江社会福利院改扩建，全市养老机构床位数超过1万张，让老年人享有老有所养、老有所依、老有所乐的幸福生活。

做好冰雪文章。深度开发“三大冰雪旅游体验区”，办好五大连池冰火欢乐季、中国？瑷珲中俄冰雪季、逊克大平台雾凇等主题活动，推出雪地飘移、冬捕、雪地摩托等娱乐项目，打造黑河冰雪旅游品牌。发展寒地试车产业，加强与国内外寒区试验场、各大院校和汽车科研院所交流合作，提高寒区汽车测试技术服务能力，做大做强国际汽车低温试验基地，启动建设黑河汽车经济产业园冬季试验场，炒热黑河冬季。

大力发展现代服务业。积极推动金融创新，吸引发达地区金融机构入驻我市，鼓励本地金融企业创新产品，支持省农业信贷担保公司在北安、嫩江设立分支机构。运营好现有市级电商平台，深化与淘宝、京东、苏宁等国内知名电商平台合作，扩大网上销售规模。完善合作区跨境电商园区功能，争取创建国家级电商示范基地。利用现代物流体系发展生活服务业，培育壮大连锁经营、同城配送等新型商贸业态，推动快递服务以城带乡、进村入户，促进城乡生活服务业快速发展。

(五)实施城市功能提升工程，建设特色鲜明的口岸城市。统筹当前与长远、地上与地下、建设与管理，一年强基础、两年上台阶、三年大提升，构筑宜居宜业宜游的品质黑河。

提升统筹规划水平。尊重自然和城市发展规律，科学规划城市空间，将老城区培育为城市商业核心区，西部城区完善为城市宜居生态区，东部城区提升为城市文化活力区，工业区优化为环保产业集聚区，桥头区打造为城市产业功能区。科学调整城市总体规划，编制滨江大道、生态保护与建设规划，加快修复城市自然生态。编制市区江河湖库连通项目，分步实施引水进城、四湖连通、月牙湖生态治理工程，启动北安山口水库引水工程，用水系景观滋养城市，打造城水相依的宜居生活空间。

提升基础设施建设水平。加快完善城市功能，重点实施“122556”工程。推进1条通道，借助国道丹阿公路市区过境段建设机遇，谋划建设东连桥头区、西通卧牛湖的滨江景观大道，打造市区沿江大通道；启动2个区域，建设黑龙江大桥桥头区、跨江空中索道区市政基础及配套设施；建设2个市场，启动石材、农机市场建设，优化城市空间布局；实施5条道路，建设龙科街延伸段、浮箱固冰通道连接段、土产路、逸夫中学北侧“断头路”、大黑河岛环路；新建5座公园，实施瑷珲—腾冲中国人口地理分界线主题公园、二道豁洛水景公园、老通江街文化长廊公园、西部休闲公园、民国特色仿古街公园建设，拓展城市公共游园空间；改造6大重点，实施老旧住宅、建筑立面、楼体亮化、道路、广场和公厕改造升级工程。推进“厕所革命”，下大气力改善城乡居民生活条件。加快市区棚户区建设，完成4个回迁区域21栋、9.5万平方米安置楼建设。发放住房租赁补贴5000户。改造市区老旧供热管网9.6公里。继续实施燃气管网入户工程，铺设天然气管线23公里，新增居民用户1万户。

提升智慧化精细化管理水平。加快智慧黑河建设，建成“一平台、两中心”，提高应急预警能力。强化土地市场供应调控和程序管理，提升土地利用价值。加强建筑市场管控，健全房地产企业准入和诚信机制，加大资信审核监管力度，合理确定房地产市场开发计划。明确责任边界，做好《黑河市市容和环境卫生管理条例》组织实施。探索完善“市区一体”城市管理机制，合理确定职责任务，将精细化管理向居民社区、背街背巷、城乡结合部及周边区域延伸。科学规划户外牌匾、广告设置，开展专项清理，营造整洁的城市视觉空间。

(六)实施“黑河蓝”保护工程，建设和谐美丽新家园。坚定实施绿色崛起战略，加大生态保护和环境治理力度，推动生态文明建设取得更大成效。

强化生态系统建设。实施绿化黑河大地行动，完成造林绿化9.38万亩、森林抚育25万亩，推进退耕还林还草还湿，恢复草原植被，建立市场化、多元化生态补偿机制，开展生态红线划定工作，守住生态环境底线。统筹全市国家级省级自然保护区、森林公园和湿地公园的保护开发，争取嫩江圈河湿地晋升国家级湿地公园。高度重视森林防火工作，继续坚持高一个标准、严一层要求、早一步发动，建立完善长效管控机制，严格落实属地责任，坚决杜绝重特大森林火灾发生。深化与阿州在跨境自然保护区、边境动物迁徙廊道等方面合作，保护好共同的生态圈。

守护优美生态环境。深入实施农业“三减”行动，推广逊克秸秆还田保护性耕作模式，秸杆还田面积900万亩以上。加大中小河流及主要支流治理力度，推进讷谟尔河、多金河等治理工程，争取国境界河岸岛防护三期工程开工。全面建立河长制、湖长制，实行最严格水资源管理制度考核，严格执行水资源开发利用控制、用水效率控制和水功能区限制纳污“三条红线”，建设天蓝、地绿、水清的美丽黑河。

全力推动绿色发展。加强矿山环境综合治理，推进煤矿废弃地和已关闭煤矿专项治理，新增耕地200公顷。加快振兴煤矿等3个国家级绿色矿山试点建设，积极发展节能环保、清洁生产、清洁能源产业，大力推广建筑节能和绿色建筑。开展创建节约型机关、绿色家庭、绿色学校、绿色社区和绿色出行行动，在全社会倡导简约适度、绿色低碳的生活时尚。

(七)实施改革攻坚推进工程，激发振兴发展动力和活力。全面落实中央和省委、市委重大改革部署，让改革更精准对接发展所需、基层所盼、民心所向。

深化重点领域改革。深入推进“三权”分置，完成省级土地确权、北安全国第二批农村集体产权制度改革试点。释放国有林场发展活力，确保完成国有林场改革任务。推进供销社综合改革，构建服务“三农”发展新平台。稳步推进财政领域改革，完善预算管理制度，制定市以下财政事权与支出责任划分改革方案，优化财政支出结构，防范地方政府债务风险。深化投融资体制改革，探索采用融资租赁、资产证券化等方式，加强与社会资本合作，争取有更多项目纳入国家和省PPP项目库。

深化行政体制改革。推进“放管服”改革，动态调整权力和责任清单，实施市场准入负面清单制度。深化商事制度改革，推进“多证合一”，提高工商注册便利化水平。加强“诚信黑河”建设，在重点领域和重点行业实施红黑名单制度。推进事业单位分类改革，推行政府购买服务，能够通过社会化用工解决的不再新设事业单位，已经设立的逐步有序退出。整合公共资源交易平台，完善监管机制，促进公共资源交易阳光操作。

深化社会事业领域改革。推动城乡义务教育一体化改革，推广嫩江义务教育学校教师“县管校用”管理改革试点经验，促进城乡、校际间师资力量均衡。启动高考综合改革，提升高中教育质量，争取高考成绩实现更大突破。深化文化体制改革，实行文化市场综合执法机构和职能整合。巩固市县两级公立医院改革成果，建立现代医院管理制度，推进新型医联体建设，开展个性化家庭医生签约服务，让群众看病不难、治病不贵。

(八)实施民生暖心工程，提升群众幸福感。始终把人民群众的利益摆在至高无上的位置，坚持在发展中保障和改善民生、补齐民生短板，实现广大群众的美好生活梦想。

激发创业创新活力。落实促进就业各项扶持政策，实施重点群体就业援助项目，拓宽农村转移劳动力就业渠道，推广黑河就业地图，确保城镇登记失业率控制在省定指标以内，实现零就业家庭动态清零。推广复制小微企业科技创业园经验，加强创业孵化基地建设，引导鼓励科技成果转化，积极培育科技型企业。加大引才引智力度，借助智库联盟、院士工作站和各类高端论坛柔性引进顶级专家团队，大力引进和培养教育、医疗、法律、电子商务等领域急需紧缺人才，为创业创新提供有力支撑。

提升公共服务能力。坚持把教育事业放在优先位置，新建改扩建公办幼儿园6所，创建义务教育标准化学校3所，完成49所薄弱学校改造和北安、嫩江、五大连池(含五大连池风景区)县域义务教育基本均衡发展建设任务，争取嫩江国家级农村职成教育示范县通过验收。深化与黑河学院全方位战略合作，市校共建文化产业园、黑河冰球馆，共同推进旅俄华侨纪念馆改扩建项目。市委党校新校区投入使用。办好老年大学。完善城乡医疗卫生服务体系，加快市疾病预防控制中心改扩建、五大连池人民医院康复中心综合楼建设，完成61所贫困村卫生所标准化建设，选拔推荐“龙江名医”。加大文化惠民力度，实施书香黑河工程，创建省级公共文化服务体系示范区，开展好“城市之光、金色田野”活动。深入实施“健康龙江行动”，积极备战省第十四届运动会，办好市第三届全民健身运动会，打造充满活力的动感城市。

提高群众生活水平。坚持兜底线、织密网、建机制，抓好全民参保登记，扩大社保参保范围，推进实施机关事业单位养老保险制度改革，继续实施失业保险稳岗补贴和技能提升补贴政策。做好城乡低保、特困供养工作。对符合条件的城乡因病致贫重特大疾病患者实施医疗救助，救助对象政策范围内住院自负费用救助比例全市平均达到70%以上。提高机关事业单位工作人员补贴水平，市直住房公积金财政匹配比例由应发工资的8%提高到10%，市直住房提租补贴比例由应发工资的6%提高到9%。

加强和创新社会治理。构建多方参与、共同治理的城乡社区治理体系，增加社区群众活动场所。严格落实安全生产责任制，加强安全防控能力建设，确保安全生产形势持续稳定。加强智慧消防建设，提升火灾防控和应急救援水平。深入开展公路运输超限超载治理，整顿道路运输市场秩序。实施信访积案和疑难案件集中化解专项行动，加强超前防控和源头治理，打造网上信访主渠道。推进平安黑河建设，加快构建立体化信息化社会治安防控体系。深入开展法治宣传，做好“七五”普法中期验收。提升国防动员和后备力量建设水平，完成民兵调整改革任务，争创省级双拥模范城“五连冠”，维护边境秩序持续稳定。同时，进一步做好侨务、妇女儿童、民族宗教、人防、防震减灾、档案、地方史志等工作。

各位代表，2018年，我们在全面推进经济社会发展的同时，集中精力、重点突破，全力抓好事关长远的“十件大事”。一是确保建档立卡的贫困村和贫困户全部脱贫。二是市区棚户区回迁安置房建设主体工程完工。三是启动城市功能三年提升工程和农村人居环境整治三年行动。四是开工建设黑龙江大桥桥头区及口岸基础设施。五是跨江空中索道开工，同步推进口岸和市政配套设施建设。六是争取跨境经济合作区、重点开发开放试验区、跨境旅游合作区和边境旅游试验区政策落地黑河。七是确定北黑铁路地方段改造升级主体和最佳合作方式。八是实施黑河瑷珲机场国际查验设施改造和机坪改扩建等项目。九是争取黑河口岸连通工程和滨江景观大道列入交通部“十三五”规划，完成开工前各项准备工作。十是保税物流中心(B型)通过验收并投入使用。对这“十件大事”，我们将明确任务、落实责任、倒排时间、全力推进，努力为黑河发展打牢根基。

做好今年工作，必须加强政府自身建设。我们要以党的十九大精神武装头脑，贯彻落实习近平总书记关于加强作风建设的重要批示精神，深入开展“不忘初心、牢记使命”主题教育，树立弘扬“三个好把式”“五个好作风”先进典型，以作风转变促进发展环境改善。深入开展“学习之冬、招商之冬、创新之冬”活动，把“五种思维”落实到谋振兴的蓝图上，把“八个本领”体现到促发展的实践中，提振干事创业精气神。主动接受市委领导，坚决贯彻市委各项部署，自觉接受人大法律监督、政协民主监督和社会监督、舆论监督，把政府工作全面纳入法治轨道。强化廉政风险防控，实现公共资金、国有资产、国有资源、领导干部经济责任审计监督全覆盖。

各位代表，我们的事业深深植根于黑河这片热土，我们的力量深深植根于广大群众。让我们在省委省政府和市委的坚强领导下，积极投身新时代中国特色社会主义伟大实践，不动摇、不松劲、不停步、不折腾，共同书写建设现代化新黑河的壮丽篇章！
